# Cooperation in brown capuchin monkeys: social tolerance, individual traits, and partner sensitivity in a Stag Hunt task

**DOI:** 10.1007/s10071-026-02107-8

**Published:** 2026-09-16

**Authors:** Kaustabh M. Baruah, Josep Call, Amanda M. Seed

**Affiliations:** School of Psychology and Neuroscience, University of St Andrews, St Andrews, UK

**Keywords:** Capuchins, Cooperation, Exploration, Partner choice, Stag Hunt, Tolerance

## Abstract

Mutualism with free partner choice is considered a key foundation in the evolution of cooperative behaviour, yet many experimental studies restrict partner choice or provide few alternative options, limiting our insight into the factors shaping cooperative decisions. We investigated cooperation in two groups of captive brown capuchin monkeys (*Sapajus* spp.) using an apparatus implementing the Stag Hunt paradigm. Individuals could freely choose between a cooperative option, a “Stag box” requiring simultaneous lever pressing by two individuals to obtain high-value rewards, and four individually operable “Hare boxes” that yielded lower-value rewards. Monkeys were free to select partners and interact with any boxes in six sessions. Most individuals (East: *N* = 22; West: *N* = 18) engaged in successful cooperative bouts in the Stag box and also obtained rewards individually from the Hare boxes. Generalized linear mixed models showed that dyadic tolerance significantly predicted the likelihood of cooperation in both groups. Individual characteristics also influenced engagement: exploratory individuals and dyads involving juveniles participated in more cooperative interactions. Further analyses of pressing behaviour indicated that the partner presence effect differed across contexts and groups. In the East Group, partner presence increased Stag-box pressing but lowered Hare-box pressing, whereas in the West Group, Stag-box pressing increased across sessions but did not differ depending on whether a partner was present or absent. These findings highlight the importance of integrating social relationships and individual traits to understand cooperative decision-making in primates and demonstrate how paradigms allowing free partner choice can reveal the social dynamics underlying cooperation.

**Supplementary Information:**

The online version contains supplementary material available at https://doi.org/10.1007/s10071-026-02107-8.

## Introduction

Many animals live in social groups because sociality provides adaptive benefits such as reduced predation risk (Foster and Treherne 1981; van Schaik 1983), increased foraging efficiency (Grueter et al. 2018) and mating opportunities (Majolo and Huang 2022). Group living also creates opportunities for cooperation, defined broadly as two or more individuals acting toward a shared goal (Boesch and Boesch 1989). Cooperative interactions can vary in cognitive demands, from simple synchrony in space and time to coordination requiring complementary roles (Boesch and Boesch 1989).

Mutualism, in which all participants obtain immediate benefits, is often considered a foundational pathway in the evolution of cooperation (De Waal 2000; Bshary and Bergmüller 2008; Tomasello et al. 2009). Because mutualistic interactions do not require individuals to forgo immediate pay-offs, they may depend primarily on the ability to coordinate activity rather than complex goal alignment. Experimental paradigms have, therefore, examined mutually beneficial cooperation by providing opportunities for individuals to obtain rewards simultaneously through coordinated action.

Experimental studies of cooperation have often used rope-pulling or loose-string tasks in which individuals must act simultaneously, wait for, or coordinate with a partner to obtain food, while acting alone results in failure (Crawford 1937; Hirata and Fuwa 2007). These approaches have been applied across multiple primate and non-primate species demonstrating that coordinated action is widespread (Mendres and De Waal 2000; Hare et al. 2007; Seed et al. 2008; Plotnik et al. 2011). However, cooperative success is not uniformly distributed across dyads within a social group and has proven highly variable (Melis et al. 2006a; Suchak et al. 2014; Molesti and Majolo 2016), prompting a shift from asking whether animals can cooperate to identifying the social and individual conditions under which cooperation emerges.

Among these conditions, tolerance, defined as the willingness to allow others proximity to food resources or apparatus, has consistently emerged as an important predictor of cooperation (Hare et al. 2007; Petit et al. 1992; Seed et al. 2008). Accordingly, cooperation often fails in chimpanzees when dominant individuals monopolise rewards or restrict access to the task (Hare et al. 2007), but increases when individuals tolerate close proximity and reduce competitive interference, as shown in hyenas and vervet monkeys (Drea and Carter 2009; Fruteau et al. 2013).

However, despite its importance, tolerance is rarely quantified directly in cooperative experiments (Jaeggi et al. 2010a). Many studies physically separate participants or use apparatuses that prevent monopolisation (Mendres and De Waal 2000; Schwing et al. 2016, 2021). Although such designs reduce overt conflict and help isolate task comprehension, they also mask natural variation in tolerance, limiting its measurable influence (Schwing et al. 2021). Evidence from rhesus macaques shows that tolerance can emerge even among non-kin, particularly within specific contexts such as consortship dyads (Dubuc et al. 2012), underscoring the importance of relationship structure. These findings highlight the need for approaches that permit natural expression of tolerance alongside other social variables.

Affiliative bond strength has also been associated with cooperative performance (Silk et al. 2003; Cronin 2012; Molesti and Majolo 2016). However, affiliation, tolerance and dominance relationships can often covary in primate social systems, making their independent contributions difficult to isolate (De Waal and Luttrell 1989; DeTroy et al. 2022; Thierry 2022). Dominance hierarchies may shape cooperation by stabilising coordination through predictable asymmetries or constraining access through monopolisation (Jaeggi et al. 2010b). Beyond dyadic social relationships, individual characteristics can also influence which animals engage with cooperative opportunities. Previous experiments suggest that exploratory individuals may be more likely to approach novel apparatuses and facilitate engagement, while sex and age class can affect partner combinations, motivation and social risk (Mendres and De Waal 2000; Hauser et al. 2003; Bergmüller et al. 2010; Scheid and Noë 2010; Molesti and Majolo 2016). Yet, these factors are rarely examined simultaneously in socially dynamic cooperative contexts.

A further limitation of much existing cooperation research is restricted partner choice. In many paradigms, individuals are tested in experimenter-assigned dyads or offered limited partner options, often outside their wider social group (Melis et al. 2006b, 2008). Such designs can constrain the expression of social preferences and prevent individuals from selecting compatible partners. Under forced pairings, cooperative failure may reflect social incompatibility rather than limited cooperative capacity, while success may be inflated when highly tolerant or bonded partners are pre-selected. Early open attempts with macaques and chimpanzees frequently failed, partly due to low tolerance and monopolizable rewards (Petit et al. 1992; Chalmeau 1994; Chalmeau et al. 1997). Although fully open-group designs that allow spontaneous partner selection remain rare, they provide an important opportunity to examine how individuals encounter, select and coordinate with potential partners within their social group. In such settings, cooperation is not restricted to experimenter-assigned dyads or to a simple decision of whether to cooperate with a pre-selected partner. Notable examples include open-group studies with baboons, Barbary macaques, chimpanzees and long-tailed macaques (Suchak et al. 2014; Molesti and Majolo 2016; Bhattacharjee et al. 2024; O’Hearn et al. 2024). In fact, recent reviews emphasise that most work still relies on dyadic designs that do not capture natural group dynamics (Williams et al. 2022). As a result, the combined influence of tolerance, affiliation, dominance and individual traits on cooperation remains underexplored under socially naturalistic conditions.

Beyond partner choice, a related question concerns whether individuals recognise the causal role of a cooperative partner. In delayed-partner tasks, some species wait appropriately before acting (Melis et al. 2006b; Plotnik et al. 2011). However, in several studies the waiting interval was gradually increased, potentially scaffolding behaviour through incremental learning rather than spontaneous recognition of interdependence (Seed and Jensen 2011). Active coordination beyond passive waiting has been examined in fewer cases, largely in chimpanzees, and it remains difficult to determine whether apparent coordination reflects partner sensitivity or simpler mechanisms such as social facilitation (Duguid and Melis 2020). This issue is further compounded when cooperation is the only route to food, because persistence at the apparatus may reflect reward expectation rather than motivation to coordinate (Tennie et al. 2016) Experimental designs that combine unrestricted partner choice with genuine non-cooperative options are, therefore, required to distinguish cooperative decision-making from conditioned persistence or facilitative effects of partner presence in a restricted space.

One paradigm that incorporates these structural features is the Stag Hunt (Skyrms 2001). In this paradigm, individuals may cooperate to obtain a high-value reward (Stag) or act alone to secure a smaller but guaranteed reward (Hare). Failure to cooperate yields no reward, whereas solitary action produces a reliable but lower payoff. When implemented with free partner choice and accessible solo alternatives, the Stag Hunt paradigm enables investigation of how dyadic social relationships and individual variation jointly shape partner choice and cooperative outcomes. This paradigm has been previously applied across several species, including humans, chimpanzees, macaques, squirrel monkeys, dogs, rats and capuchin monkeys (Bullinger et al. 2011; Brosnan et al. 2011, 2012; Parrish et al. 2014; Duguid et al. 2014; Vale et al. 2019; Donovan et al. 2020; Robinson et al. 2021; Martínez et al. 2024; Wallner Werneck Mendes et al. 2024), where subjects appear to reach the cooperative outcome through different mechanisms, including visual matching, sensitivity to partner behaviour, leader–follower dynamics, reward-biased responding, or local/stimulus enhancement. However, these Stag Hunt studies have focused on economic decision making (examining if individuals could switch between individual and cooperative tasks according to their respective pay-offs) using computer-based, token-mediated, or apparatus-based tasks with preselected dyads, assigned partners, or restricted partner choice. To our knowledge, studies allowing individuals to freely choose their partners while retaining access to viable solo alternatives remain limited, despite being particularly valuable for examining how cooperation competes with individual strategies. We, therefore, took inspiration from the Stag Hunt paradigm in the design of this study, in that individuals could choose to work alone for small rewards, or together for greater rewards. However, it should be noted that unlike previous studies, this was not designed to examine how individuals decided to cooperate based on pay-off structure.

We investigated cooperative behaviour in captive brown capuchin monkeys (*Sapajus* spp.) using an open-group Stag Hunt paradigm. Capuchins are socially tolerant, form coalitions and engage in affiliative interactions, making them suitable for examining socially structured cooperation (De Waal 2000; Perry 2011). Individuals could freely choose between Hare boxes, operable by a single individual for low-quality rewards, and a Stag box requiring simultaneous pressing by two individuals facing each other at two spatially separated locations to obtain high-quality rewards. Partner choice was unrestricted within the group.

We addressed three primary questions regarding cooperative behaviour in capuchin monkeys. First, we tested whether capuchins, which have previously cooperated in both classic coordination tasks and Stag Hunt paradigms, (Chalmeau et al. 1997; Mendres and De Waal 2000; Visalberghi et al. 2000), would cooperate in a more open Stag Hunt framework requiring spatially separated, simultaneous coordination with free-partner choice. Second, we examined whether social factors such as tolerance and affiliative bond strength and individual traits including sex, age class and temperament predicted cooperative outcomes. Given prior evidence (Hauser et al. 2003; Majolo et al. 2006; Hare et al. 2007; Scheid and Noë 2010; Massen et al. 2015; Asakawa-Haas et al. 2016; Bhattacharjee et al. 2024), we predicted that dyads characterised by higher tolerance and stronger affiliative bonds would cooperate more frequently, and that individual characteristics reflecting life-history stage or personality would further influence participation. Third, we assessed whether individuals were sensitive to partner presence in a context where cooperation was optional. If capuchins recognise the role of a partner, pressing should increase in the presence of a partner at the Stag box, where joint action is required. Such context-dependent modulation would indicate sensitivity to the cooperative requirements of the task. To our knowledge, this is the first study to implement a Stag Hunt paradigm in nonhuman primates within an open-group setting allowing unrestricted partner choice.

## Methods

### Subjects

We tested two captive groups of brown capuchin monkeys (*Sapajus* spp.) housed at the Living Links Centre in Edinburgh Zoo. The East Group comprised 22 individuals (12 males, 10 females; 16 adults, four juveniles and two infants). The West Group comprised 18 individuals (10 males, eight females; all adults). Age class and sex were determined from zoo management records. Both groups were housed in separate open enclosures of similar sizes (~ 900 m^2^) and did not interact. Both enclosures were furnished with trees and ropes that provided vertical space as well as natural vegetation, rocks and boxes that offered shelter from adverse climatic conditions.

All individuals were present during testing sessions and were free to participate in the experiment. Participation was voluntary, and some individuals chose not to interact with the apparatus in certain sessions. Dyadic analyses included only individuals observed interacting with the apparatus at least once during the cooperation phase.

### Apparatus

**Fig. 1:**
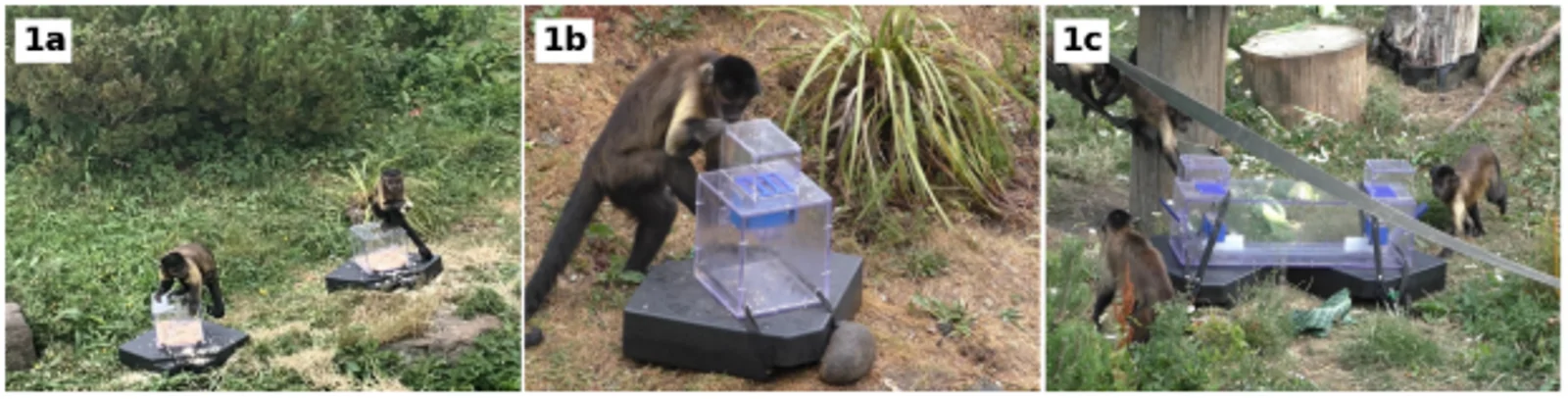
Experimental apparatus used across the three phases of the study. Figure 1a (Tolerance Box): Two food boxes presented simultaneously to quantify dyadic tolerance through spatial co-feeding under clustered food conditions. Figure 1b (Hare Box): Individual apparatus allowing a single monkey to obtain a low-value reward by independently depressing a lever. Figure 1c (Stag Box): Cooperative apparatus requiring two individuals to simultaneously depress levers at opposite ends to obtain high-value rewards

Two pairs of identical transparent polycarbonate boxes (23 × 20 × 29 cm) were used to assess tolerance (Fig. 1a). Boxes were filled with a mixture of food items (pellets, sunflower seeds, flaked maize and peanuts) and non-edible substrate (hay and sawdust) to prolong foraging time. The monkeys could put their hand directly into the open-top box to access the food within the box. Boxes were secured to water weights and anchored to the ground using tent pegs to prevent displacement.

The Hare apparatus consisted of a transparent polycarbonate box (23 × 20 × 29 cm) fitted with a lever connected to a food container (Fig. 1b). Full depression of the lever elevated the container, allowing subjects to access food through a grid placed at the top of the box. Rewards included raisins, peanuts and maize flakes. Boxes were secured and anchored as above. The apparatuses were baited at the beginning of each 1-h session with the intention of maintaining food availability throughout the testing period, and no additional food was added during sessions. Although food depletion was not quantitatively measured, small amounts of food consistently remained in the Hare box containers at the end of sessions, despite the majority of rewards being consumed during testing.

The Stag apparatus was a larger transparent polycarbonate box (95 × 20 × 29 cm) with two levers positioned at opposite ends, each connected to a food container (Fig. 1c). Simultaneous pressing of both levers was required to elevate both containers and access the reward. An internal partition prevented access when only one lever was pressed. High-value rewards (apple slices, grapes and raisins mixed with peanut butter) were used. The apparatus was secured in the same manner as the Hare and tolerance boxes. While food depletion was not formally quantified, rewards in the Stag apparatus were consumed more extensively across sessions as multiple dyads began cooperating successfully, although small amounts of food typically still remained by the end of testing. All reward types and quantities were approved by zoo staff to ensure nutritional requirements were maintained. Reward value was determined in consultation with zoo staff based on their knowledge of the capuchins’ routine feeding preferences.

### Study design and procedure

We conducted the study between February and August 2022, combining systematic behavioural observations with controlled experimental testing.

Throughout this period, we collected affiliative proximity data using scan sampling at 15 min intervals (Altmann 1974). We conducted 30 observational sessions, each comprising 6–8 scans. During each scan, we recorded the nearest neighbour of every visible individual once. We coded dyads within arm’s reach as 1 and dyads beyond that distance as 0. These proximity observations captured affiliative co-presence/proximity across non-competitive contexts, including grooming, huddling, resting in contact and close spatial association during routine activity. We used these proximity data to quantify social bond strength.

We organised the experimental component into three consecutive phases. Each phase consisted of six sessions per group, resulting in 18 sessions per group across the study. We initiated a new phase only after both groups completed all six sessions of the preceding phase. We conducted experimental sessions twice per week, with two sessions per testing day. Each session consisted of a single trial lasting 1 h. The first session ran from 11:30 to 12:30 h, and the second from 14:30 to 15:30 h.

For each session, zookeepers installed the apparatus at predetermined locations within the enclosure. All individuals had unrestricted access to the apparatus for the full 1-h period, after which zoo staff removed the boxes. Monkeys were free to approach, manipulate or leave the apparatus at any time during the session.

### Experimental phases

#### Phase 1 (Tolerance test):

In the first phase, we assessed social tolerance by measuring co-feeding behaviour. We installed two pairs of tolerance boxes (Fig. 1) simultaneously within the enclosure at standardised distances so that multiple individuals could access food at the same time. This arrangement allowed us to observe whether individuals fed alongside specific partners when food was spatially clustered.

Across sessions, we systematically varied the distance between the two boxes within each pair to examine how proximity influenced co-feeding patterns. We used three standardized inter-box distances: 2.0 m, 1.5 m and 0.8 m, the latter corresponding to approximately twice the average body length of the capuchins. We conducted two sessions at each distance and presented them in ABC–CBA order to balance potential order effects and maximise dyadic representation.

In addition to co-feeding patterns, we recorded each individual’s latency (seconds) to approach within 15 cm of a tolerance box upon its first introduction in this phase. This measure allowed us to capture initial behavioural responsiveness to the apparatus under socially competitive feeding conditions.

#### Phase 2 (Temperament test):

In the second phase, we introduced the Hare apparatus to assess individual temperament. Following completion of the tolerance phase, we presented four baited Hare boxes simultaneously within the enclosure. Providing multiple boxes reduced the likelihood that a single individual could monopolise access and created a setting in which monkeys could obtain smaller rewards independently, without requiring coordination.

Individuals were free to approach and interact with the apparatus at any time during the session. We did not provide prior training on the operation of the Hare box. Instead, we allowed subjects to discover the lever mechanism through exploration and trial-and-error learning, as is common in comparable experimental paradigms (e.g. Molesti and Majolo 2016). We considered an interaction successful when an individual fully depressed the lever and retrieved food from the elevated container. We recorded each individual’s latency (seconds) to approach within 15 cm of a Hare box upon its first introduction to quantify temperament.

#### Phase 3 (Cooperation test):

In the final phase, we tested cooperation using a Stag Hunt paradigm. After completing the temperament phase, we introduced one baited Stag box containing high-value rewards alongside four baited Hare boxes containing lower-value rewards. While the exact distances between the Stag and Hare apparatuses were not recorded, the Stag apparatus was positioned approximately centrally within the enclosure, and the four Hare boxes were placed around it towards different sides of the enclosure so that each Hare box was approximately equidistant from the Stag apparatus, with boxes separated by approximately 3 m or more based on the experimental layout. Monkeys were free to approach and interact with any apparatus during the session. In addition, zoo staff provided a low-value scatter-feed throughout the enclosure during sessions to reduce competitive crowding around the apparatus and maintain normal foraging dispersion

This arrangement provided individuals with a choice: they could act alone to secure a smaller but guaranteed reward from a Hare box or coordinate with a partner to obtain a higher-value reward from the Stag box. By offering both options simultaneously and allowing unrestricted partner choice, the design enabled us to identify which dyads engaged in joint action and whether individuals adjusted their behaviour in response to a partner’s presence.

We conducted six sessions per group in each of the three phases. In every phase, each session comprised a single continuous 1-h open-access period during which individuals could freely approach the apparatus and engage in multiple interactions.

### Behavioural recording and coding

We continuously video-recorded all interactions with the apparatus. We recorded sessions from an elevated viewing platform using three tripod-mounted video cameras and two pan–tilt–zoom (PTZ) webcams positioned to ensure full coverage of the apparatus area.

In total, we recorded approximately 60 camera-hours of footage during the Cooperation test phase across both groups. Five cameras filmed in parallel during 12 sessions (approximately 1 h each), and this total reflects cumulative recording time across cameras rather than sequential session duration.

We subsequently coded behaviour from the recordings using BORIS (Behavioural Observation Research Interactive Software, version 2023; Friard and Gamba 2016). We defined behavioural categories a priori (Table 1) to ensure consistent classification across sessions and groups.

**Table 1:** Ethogram of behavioural categories coded during experimental sessions. Definitions and spatial–temporal criteria used to classify lever-directed behaviour and proximity to the apparatus. All behaviours were recorded under both partner-present and partner-absent conditions

Behaviour	Definition	Criteria
Touch/Touching the Lever	Contact or brief hold on the lever/pedal without full upward movement of the food container	- Contact with the lever/pedal for more than 3 s - Food container moves up only halfway or remains still - If the container comes down in less than 1 s, it is still considered a ‘Touch’
Press/Pressing the Lever	Full downward push on the lever/pedal resulting in the complete upward movement of the food container	- Lever/pedal is fully pressed with one or both hands - Food container touches the top of the box - The upward movement lasts at least 1 s
Presence	Individual is within 15 cm of the apparatus for a sustained period	- Presence within 15 cm radius of the apparatus - Duration is at least 3 s

We defined “cooperation” as the simultaneous depression of the two levers at opposite ends of the Stag box such that both food containers reached the top of the apparatus. An individual’s first cooperation corresponded to the first successful completion of the cooperative task.

We defined a “cooperation bout” as a distinct successful episode beginning when both individuals pressed the levers simultaneously and ending when either individual fully released the lever for more than 3 s. This release criterion distinguished sustained disengagement from brief grip adjustments. A single bout could include one or multiple food retrievals.

We selected spatial and temporal thresholds to standardise coding and distinguish intentional task engagement from incidental proximity or accidental contact. The 15 cm radius was selected to represent entry into the immediate manipulation space of the apparatus, approximating the distance within which capuchins can directly operate the lever. The 3-second duration criterion differentiated sustained engagement from brief passing movements, while the 1-second requirement for full container elevation ensured that only complete lever actions were coded as successful presses. Together, these criteria ensured consistent and conservative classification of proximity, manipulation, and coordinated action across sessions and groups.

Video recordings were also screened for visible communicative behaviours directed towards partners during cooperative interactions, including gaze alternation, gestures, body orientation, approach signals, and recruitment-like behaviours previously reported in coordination tasks (Leavens et al. 2005; Melis et al. 2006b). Vocalisations were not systematically analysed because audio quality and enclosure-wide background noise did not allow calls to be reliably attributed to specific individuals.

To assess reliability, a second observer independently coded 20% of the videos using the complete behavioural coding scheme applied to the raw BORIS data. Inter-observer agreement was high (Cohen’s κ = 0.75 at coding onset; κ = 0.82 at completion), indicating stable coding reliability without evidence of systematic drift.

### Derived social and individual variables

#### Social factors

We quantified affiliative association (hereafter affinity) from scan-sampling proximity data collected during routine observations. For each dyad, proximity scores were aggregated across scans within each sampling day and averaged to generate a daily association value. We then calculated the mean of these daily values across all 30 sampling days to obtain a cumulative proportional association index.

Affinity scores ranged from 0 to 0.079 in the East Group (mean ± SE = 0.001 ± 0.0001) and from 0 to 0.488 in the West Group (mean ± SE = 0.071 ± 0.007), with 0 indicating no observed close proximity during the sampling period. Although the metric and calculation procedure were identical across groups, affinity values were higher in the West Group, likely because the group was undergoing a period of hierarchical restructuring during the study, resulting in increased clustering, following behaviour, alliances, and close spatial associations around particular individuals.

We calculated tolerance independently using the structured tolerance experiment. Dyads co-feeding from the same box received the highest score (4), whereas dyads co-feeding at 2 m received the lowest score (1). When more than two individuals co-fed, all possible dyads were scored. Dyads involving individuals never recorded during the tolerance sessions were excluded. Although each session involved a single distance condition between the two boxes, a dyad could be observed co-feeding multiple times within that session, either from the same box or from the two separate boxes at the session-specific distance. Scores from these co-feeding observations were averaged within each session to obtain a session-level tolerance value. If both individuals were present but the dyad was not observed co-feeding during that session, it received a session-level score of 0. We then calculated the mean of these session-level values across all six sessions to derive a final dyadic tolerance score. Tolerance ranged from 0.166 to 3.112 in the East Group (*N* = 140 dyads; mean ± SE = 1.53 ± 0.076; SD = 0.90; median = 1.67; IQR = 0.83–2.25). In the West group, tolerance scores ranged from 0.400 to 3.030 (*N* = 134 dyads; mean ± SE = 1.37 ± 0.051; SD = 0.59; median = 1.40; IQR = 1.00–1.80).

Affinity and tolerance were not significantly correlated in the East Group (Kendall’s τ = 0.048, *p* = 0.455), whereas a moderate positive association was observed in the West Group (τ = 0.267, *p* = 0.001). Variance inflation factor diagnostics indicated no problematic multicollinearity (all VIF < 2). Conceptually, affinity captures baseline affiliative association during routine activity, whereas tolerance reflects spatial permissiveness under competitive feeding conditions. We therefore retained both as distinct predictors in subsequent analyses.

#### Individual factors

Individual-level predictors included temperament (operationalised as exploratory tendency), age class and sex. We measured temperament behaviourally as latency (s) to approach the experimental apparatus at the start of a session. Approach was defined as coming within 15 cm of a Hare and Tolerance box for the first time. Latency to approach is widely used in comparative research as a behavioural proxy for exploratory tendency and neophobia in novel task contexts (Kendal et al. 2005; Gilby et al. 2008; Scheid and Noë 2010).

In this study, exploratory tendency was defined as latency to approach the apparatus, with shorter latencies indicating higher exploratory tendency. This measure reflects reduced neophobic hesitation rather than a stable personality trait or active novelty seeking. Individuals classified as more exploratory were not necessarily more attracted to the apparatus, but may have been less avoidant when encountering it. Thus, exploratory tendency here captures context-specific tolerance of novelty rather than generalized risk-taking. As individuals were tested in their social groups, this measure may also have been influenced by social context, including rank-related access or inhibition near the apparatus.

To derive an individual exploratory index, we rank-ordered first-approach latencies separately for each apparatus and group (shortest latency = highest rank) and calculated the mean rank across the Hare and Tolerance boxes. Higher scores therefore indicate individuals that approached the apparatus more readily in this task context. The resulting index ranged from 0 to 19.5 in the East Group (mean ± SE = 11.41 ± 1.23) and from 2.5 to 16.5 in the West Group (mean ± SE = 9.50 ± 1.47).

Differences in engagement are theoretically relevant because individuals that approach the apparatus more readily are more likely to be present when coordination opportunities arise, thereby increasing their potential participation in cooperative interactions.

### Statistical analyses

All analyses were conducted in R Studio (R version 4.2.3) using the packages *lme4* (Bates et al. 2015), *glmmTMB* (Brooks et al. 2017), *MASS* (Venables and Ripley 2002), *car* (Fox and Weisberg 2018), *DHARMa* (Hartig 2016), and *emmeans* (Lenth 2023). Descriptive statistics and selected supplementary tests were performed in JASP (version 2023) . Figures were generated using ggplot2 (Wickham 2016).

Model diagnostics were performed for all models. Overdispersion and residual structure were evaluated using the simulateResiduals(), testDispersion(), and testZeroInflation() functions in *DHARMa.* Multicollinearity was assessed using variance inflation factors via the vif() function in *car* that indicated no problematic multicollinearity (East Group: tolerance = 1.70, affinity = 1.70; West Group: tolerance = 1.27, affinity = 1.27). All VIF values were well below the commonly accepted threshold of 5, and both predictors were therefore retained in the models. Candidate model families were compared using Akaike Information Criterion (AIC) and residual diagnostics. Predictor contributions were evaluated using likelihood-ratio tests (LRTs) comparing full models with reduced models where appropriate.

To check whether social factors predicted the likelihood of a dyad to cooperate, dyadic-level analyses were conducted using a Generalized Linear Mixed Model (GLMM) with a binomial error distribution and logit link function, fitted using the glmer() function in the lme4 package (Bates et al. 2015). The binary dependent variable indicated whether a dyad cooperated at least once during the experimental period. Each potential dyad (restricted to dyads in which at least one of the two individuals was observed during the cooperation test) was included as a data point. Dyads involving individuals that were never observed were excluded from the analysis, whereas dyads that were observed but never cooperated were retained and coded as 0.

To partially account for non-independence arising from individuals appearing in multiple dyads, we included crossed random intercepts for the identities of both dyad members (ID1 and ID2). This structure accounts for repeated occurrence of individuals within each dyadic position; however, because dyadic positions were not biologically distinct and individuals could occur as either ID1 or ID2 across different dyads, individual identity was not represented by a single random effect across both positions. The combined model included dyadic tolerance, affiliative bond strength (affinity), group identity, and the interactions between each social predictor and group. This model tested whether tolerance and affinity predicted cooperative success across the complete dataset and whether their effects differed between the East and West groups. Tolerance and affinity were mean-centred and standardised across the combined dataset before analysis to improve model stability and facilitate interpretation. Predictor contributions were assessed using likelihood-ratio tests (LRTs) comparing the full model with hierarchically reduced models. The contribution of each social predictor was evaluated by removing its main effect and corresponding interaction with group while retaining all remaining fixed effects and the crossed random intercepts. Interaction terms were tested by removing each interaction individually while retaining the constituent main effects, and their joint contribution was assessed by comparing the full model with an additive model containing tolerance, affinity, and group identity but no interaction terms. Overall model fit was assessed by comparing the full model with a null model containing only the crossed random intercepts, and with a group-only model to determine whether the social predictors explained cooperative success beyond differences between the East and West groups.

To examine whether individual characteristics predicted cooperative participation, we fitted individual-level negative binomial GLMs using the glm.nb() function from the MASS package. The response variable was the total number of cooperative bouts in which each individual participated, calculated by summing all successful dyadic interactions involving that individual across sessions. Exploratory tendency, age class and sex were included as predictors in the East group, whereas only exploratory tendency and sex were included in the West group because all West-group individuals were adults. We fitted negative binomial models with a log link function because Poisson models showed substantial overdispersion. Individual-level models were retained separately for the two groups because an equivalent biologically meaningful predictor structure could not be fitted across both groups. The East group contained adults, juveniles, and infants, whereas the West group consisted entirely of adults. Age class could, therefore, be estimated only within the East group and was structurally confounded with group in a combined analysis. The separate models were consequently interpreted as within-group analyses of individual predictors, and differences between their coefficients were not treated as formal statistical evidence of between-group differences. Predictor contributions were evaluated using LRTs comparing the full model with reduced models in which each predictor was removed while retaining the remaining predictors. As a supplementary analysis, we also tested whether exploratory tendency predicted overall task engagement, defined as the total number of press and touch behaviours directed towards all experimental boxes within a session. As exploratory index was derived separately within each group and the groups differed in demographic composition, these supplementary analyses were also fitted separately for the East and West groups using negative binomial GLMMs, with exploratory tendency and session as fixed effects and subject identity as a random intercept.

We then tested whether categorical dyad compositions (age and sex categories) were associated with cooperative success, we conducted Pearson’s chi-square tests comparing the distribution of successful and unsuccessful dyads across dyad types. Where expected cell frequencies were below 5, Fisher’s exact tests were used. Effect sizes were calculated using Cramér’s V. These analyses complemented the mixed-model approach by testing whether cooperative outcomes were non-randomly distributed across dyad categories.

Finally, we analysed whether individuals adjusted their behaviour depending on the presence of a partner and whether this effect differed across task contexts and groups, counts of Press behaviour were analysed across partner-present and partner-absent conditions for both the Stag and Hare boxes. Press counts were analysed using Generalized Linear Mixed Models (GLMMs) fitted with the glmmTMB() function (Brooks et al. 2017). The unit of analysis was the individual within each session. As individuals were observed repeatedly across sessions and experimental contexts, subject identity was included as a random intercept to account for non-independence of repeated observations. The fixed-effect structure of the global model included partner presence (present vs. absent), box type (Hare vs. Stag), group identity (East vs. West), and session, together with their interaction terms. Session was treated as a continuous covariate to capture potential linear changes in behaviour across repeated trials, reflecting possible learning or increasing familiarity with the apparatus. Competing model families (Poisson, negative binomial, and zero-inflated negative binomial) were compared using Akaike Information Criterion (AIC) and residual diagnostics implemented in the DHARMa package (simulateResiduals(), testDispersion(), testZeroInflation(), and the best-fitting distribution was retained for inference. Estimated marginal means were subsequently calculated using the emmeans package (Lenth 2023) to interpret significant interaction effects and evaluate partner-presence effects within each combination of box type and group.

The global model explicitly tested our primary comparative question of whether the effect of partner presence on pressing behaviour differed across task contexts and groups, with session included as a covariate to account for general linear change across repeated testing. We then conducted secondary follow-up analyses to examine whether the relationship between partner presence and pressing changed across sessions within each specific group-by-task context. Separate models were fitted for each group and task context, with partner presence, session and their interaction entered as fixed predictors and subject identity included as a random intercept. Model families (Poisson, negative binomial, and zero-inflated negative binomial) were compared using AIC and DHARMa diagnostics, and the best-fitting distribution was selected for each subset analysis. These follow-up models were used to describe context-specific temporal trajectories of partner-associated pressing and were not treated as independent primary tests of group or task differences.

## Results

**Fig. 2:**
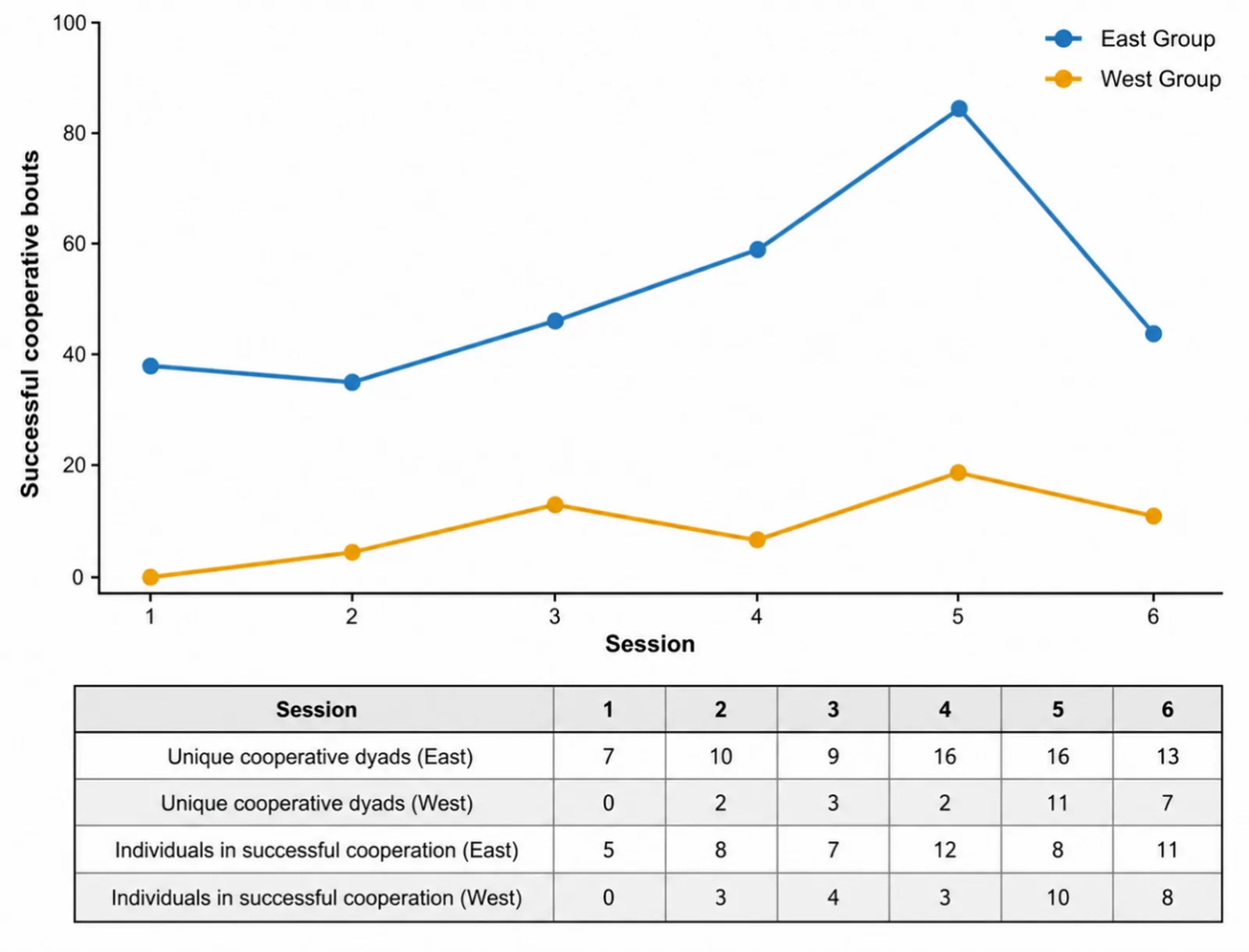
Number of cooperative bouts across sessions in the East and West groups. Lines represent the total number of successful cooperative bouts observed in each session (1–6) for each group. The East Group exhibited consistently higher cooperative frequencies across sessions, with cooperation emerging from Session 1, whereas cooperation in the West Group began in Session 2 and remained comparatively lower overall. Values reflect total counts per 1-h session. The accompanying table reports the number of unique cooperative dyads and the number of individuals participating in successful cooperation in each session for both groups

Capuchins successfully cooperated in the Stag box in both groups (Fig. 2). Across the experiment, a total of 306 cooperative bouts were recorded in the East group and 54 bouts in the West group. Cooperation emerged earlier and occurred more frequently in the East group. Successful cooperative bouts were observed from the first session in the East group, whereas cooperation began in session 2 in the West group (Fig. 2). Individual participation in successful cooperation was uneven in both groups, as expected in a voluntary group-testing design. In the East group, 14 of 22 individuals participated in at least one successful cooperative bout, with individual participation ranging from 0 to 143 bouts. In the West group, 13 of 18 individuals participated in at least one successful cooperative bout, with individual participation ranging from 0 to 27 bouts. Thus, successful cooperation was not restricted to a single dyad or individual, although some individuals contributed disproportionately more than others.

**Fig. 3:**
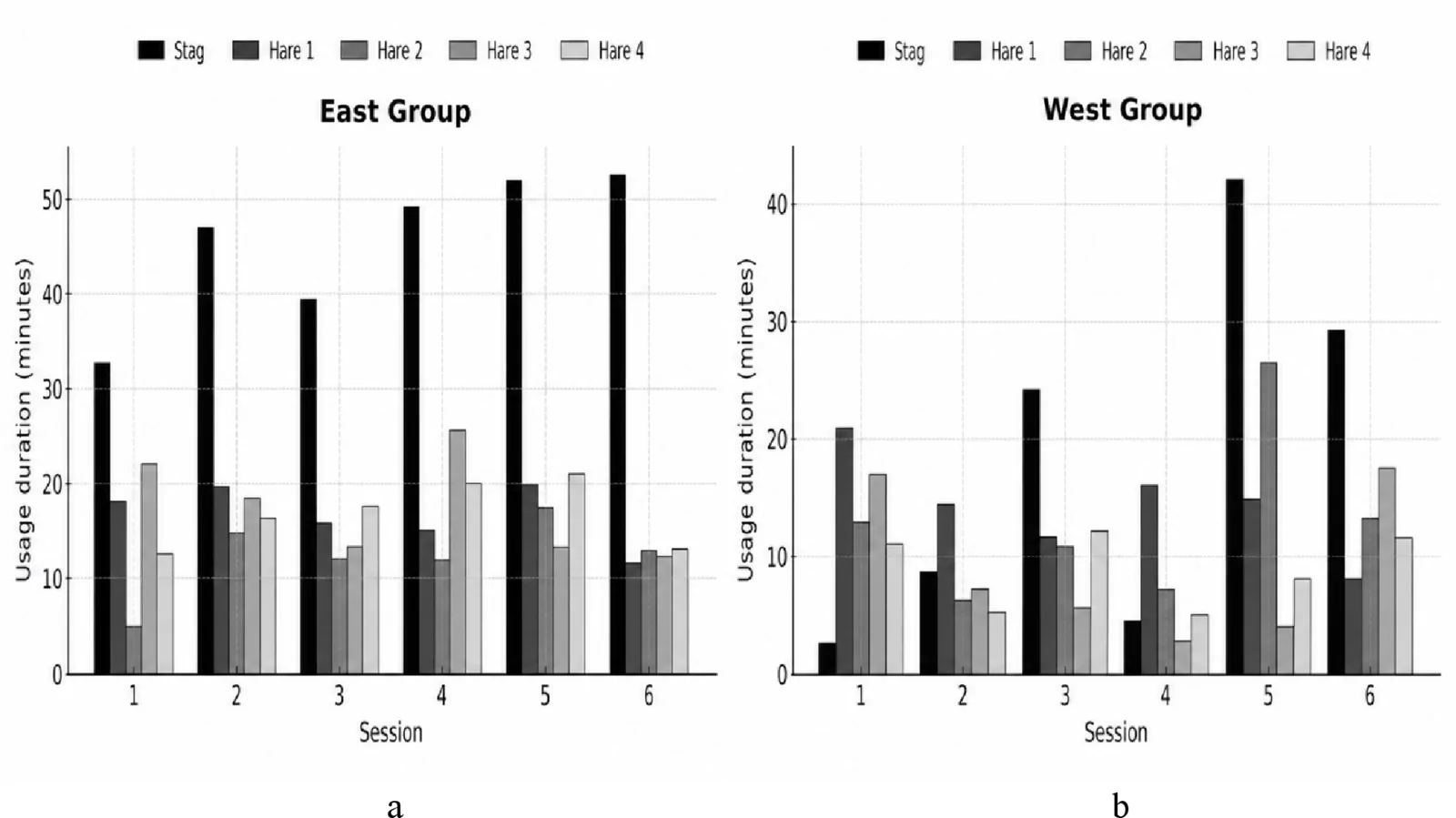
Utilisation of the Stag box and four Hare boxes across sessions in the East and West groups. Bars show the cumulative duration, in minutes, for which group members interacted with each box during each session, calculated as press duration plus touch duration. Durations were recorded at the individual level and summed across individuals within each session. Thus, if two individuals interacted with the same box simultaneously for one minute, this contributed two individual-minutes to the plotted value. Values represent cumulative group-level utilisation of each box and were not averaged across individuals. Panels show the East group (3a) and West group (3b)

Cumulative participation curves further showed that new successful individuals and dyads continued to emerge across sessions in both groups (Fig. S1). This indicates that Stag box participation and successful cooperation had not clearly reached an asymptote within the six test sessions. Both groups interacted with the Stag box and all four Hare boxes, which were available simultaneously in the enclosure. Across sessions, the cumulative duration of utilisation of the Stag box/ engagement with the Stag box was generally greater than that of any individual Hare box in both groups (Fig. 3). Stag-box utilisation/engagement was already substantial in Session 1 in the East group, whereas it was initially limited in the West group and increased over subsequent sessions.

### Social predictors of cooperative success

**Fig. 4:**
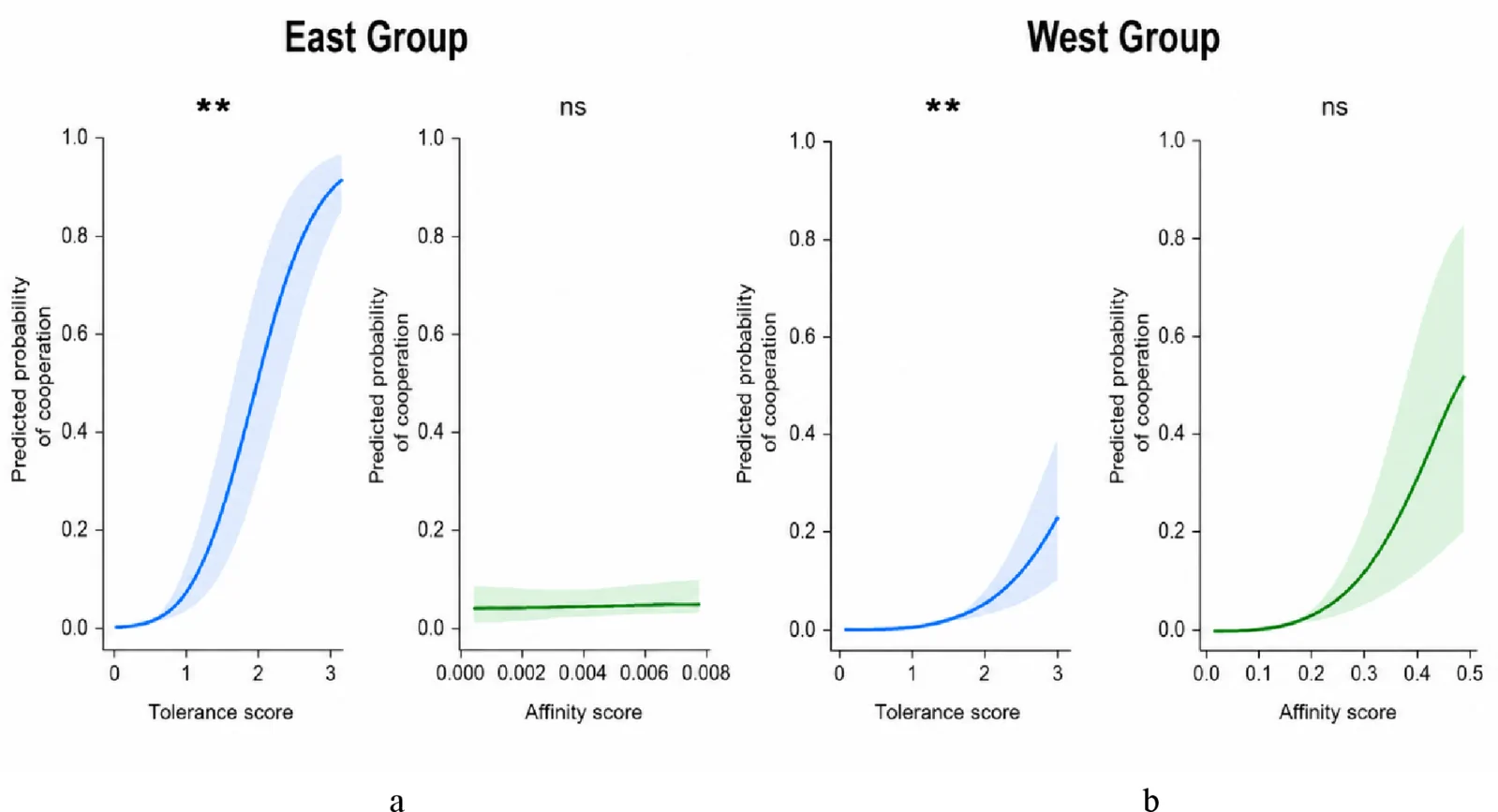
Social predictors of dyadic cooperation in capuchin monkeys. (**a**) East group. (**b**) West group. Model-predicted probability of dyadic cooperation as a function of dyadic tolerance and affinity scores. Solid lines represent predictions from the combined binomial generalized linear mixed model, with shaded ribbons indicating 95% confidence intervals. Predictions are shown across the observed range of each predictor within each group, with the other continuous predictor held at its mean. Tolerance and affinity were mean-centred and standardised across the combined dataset before analysis, although raw predictor values are displayed on the x-axes. ** indicates *p* < 0.05; *ns* indicates a non-significant relationship

Greater dyadic tolerance significantly predicted a higher likelihood of cooperation across both groups (Fig. 4; Table S1). In the combined model, tolerance made a significant overall contribution to cooperative success (χ²(2) = 18.49, *p* < 0.001), with greater tolerance associated with an increased probability of cooperation in the East reference group (β = 1.70 ± 0.53 SE, z = 3.18, *p* = 0.001). The tolerance × group interaction was not significant (χ²(1) = 0.17, *p* = 0.677), indicating that the strength of this positive association did not differ significantly between the East and West groups.

In contrast, affiliative bond strength did not significantly predict cooperative outcomes overall (χ²(2) = 5.19, *p* = 0.075). Affinity was not significant in the East reference group (β = 12.50 ± 18.84 SE, z = 0.66, *p* = 0.507), and the affinity × group interaction was also non-significant (χ²(1) = 0.39, *p* = 0.535), providing no evidence that its association with cooperation differed between groups. Group identity itself did not significantly predict cooperative success (β = −7.98 ± 8.93 SE, z = − 0.89, *p* = 0.372).

The full model significantly improved fit relative to the random-effects-only null model (χ²(5) = 35.55, *p* < 0.001). Adding tolerance and affinity to a model containing group identity also significantly improved model fit (χ²(2) = 32.68, *p* < 0.001), whereas removing both group interactions did not reduce model fit (χ²(2) = 0.67, *p* = 0.714). Variance components from the combined model indicated substantial heterogeneity attributable to the identities of both dyad members (Var_ID1 = 2.05, Var_ID2 = 4.89).

### Individual predictors of cooperative participation

**Fig. 5:**
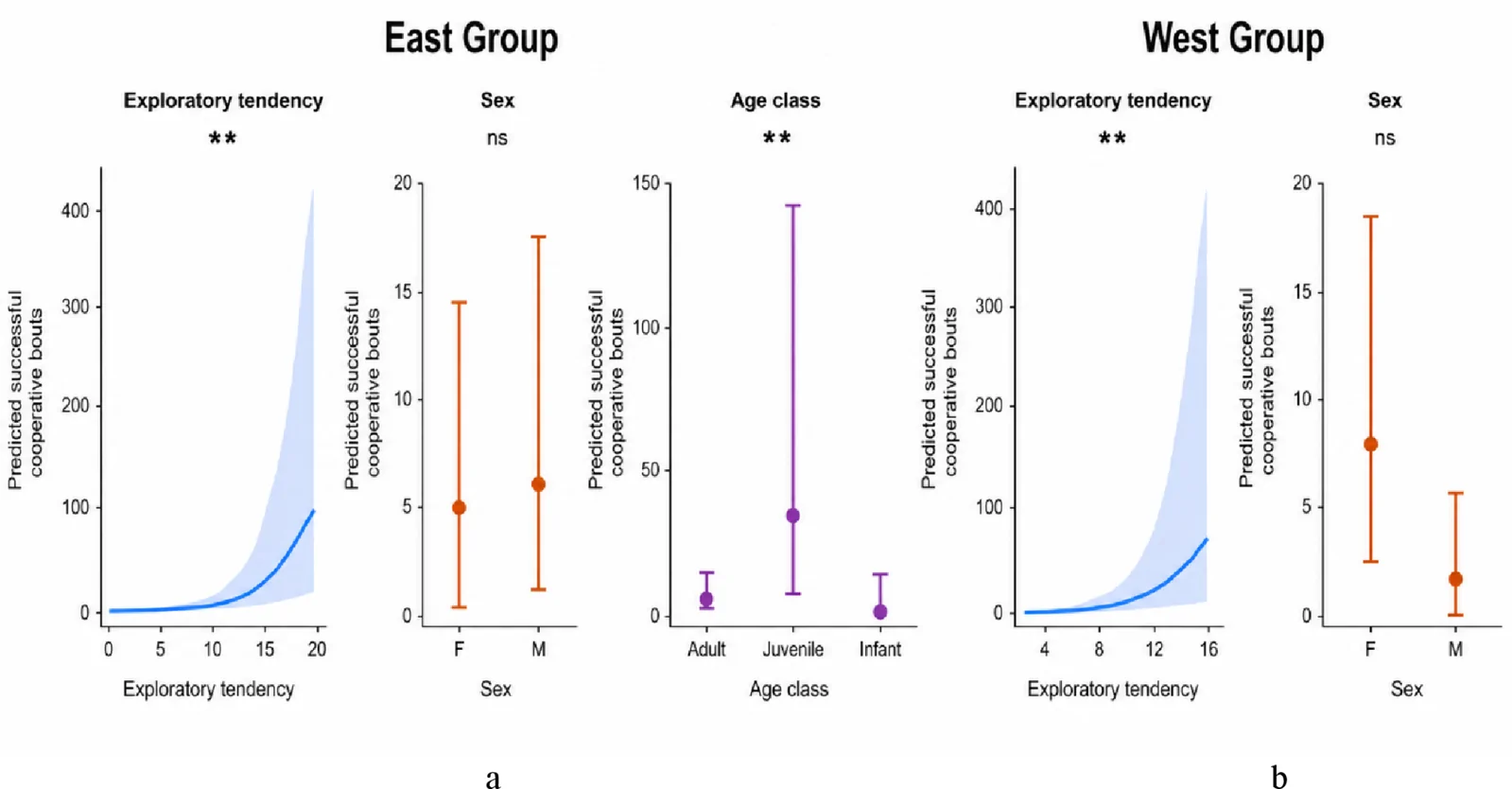
Individual predictors of dyadic cooperation in capuchin monkeys. (**a**) East group. (**b**) West group. Model-predicted probability of dyadic cooperation as a function of exploratory tendency, sex and age class. Solid lines represent predictions for exploratory tendency, with shaded ribbons indicating 95% confidence intervals, while points and error bars represent estimated probabilities and 95% confidence intervals for categorical predictors. Predictions are shown across the observed range of exploratory tendency within each group, with the remaining predictors held at their reference levels or means as appropriate. Separate negative binomial models were fitted for each group because age class was represented only in the East group. ** indicates *p* < 0.05; *ns* indicates a non-significant relationship

Individual exploratory tendency significantly predicted participation in cooperative bouts in both groups (Fig. 5, Table S2). In the East group, exploratory tendency positively predicted cooperative participation after accounting for age class and sex (β = 0.344 ± 0.076 SE, z = 4.52, χ²(1) = 12.33, *p* < 0.001), corresponding to a 41% increase in expected cooperative counts per unit increase in exploratory tendency. Age class also significantly predicted cooperative participation after accounting for exploratory tendency and sex (χ²(2) = 6.75, *p* = 0.034). Juveniles participated in significantly more cooperative bouts than adult females (β = 1.881 ± 0.770 SE, z = 2.44, *p* = 0.014), whereas infants did not differ significantly from adult females. Sex did not significantly predict participation after accounting for exploratory tendency and age class (χ²(1) = 0.13, *p* = 0.722). In the West group, exploratory tendency again significantly predicted cooperative participation after accounting for sex (β = 0.313 ± 0.093 SE, z = 3.38, χ²(1) = 7.63, *p* = 0.006), corresponding to a 37% increase in expected cooperative bouts per unit increase in exploratory tendency. Sex did not significantly predict participation after accounting for exploratory tendency (β = −1.143 ± 0.769 SE, z = − 1.49, χ²(1) = 1.97, *p* = 0.161).

In the supplementary task-engagement analysis, exploratory tendency positively predicted overall engagement counts in both groups (Table S3). In the East group, a one-standard-deviation increase in exploratory tendency was associated with a 3.77-fold increase in expected engagement counts (β = 1.33, SE = 0.32, χ²(1) = 14.37, *p* < 0.001). In the West group, the corresponding increase was 1.77-fold (β = 0.57, SE = 0.23, χ²(1) = 5.58, *p* = 0.018; Table S3).

Dyadic age composition significantly predicted cooperative success in the East group (χ²(5) = 31.31, *p* < 0.001; Fisher’s exact *p* < 0.001; Cramér’s V = 0.43). Juvenile–juvenile and adult–juvenile dyads exhibited the highest within-category success rates. In contrast, the West group consisted exclusively of adult individuals and therefore contained only adult–adult dyads, which showed a within-category success rate of 12.69%. Dyadic sex composition did not significantly predict cooperative success in either group (East: χ²(2) = 1.89, *p* = 0.389; West: χ²(2) = 1.17, *p* = 0.558; Fisher’s exact tests ns).

### Sensitivity to the partner’s presence: press behaviour across task contexts

Press counts were analysed to determine whether individuals adjusted their engagement with the apparatus depending on the presence of a partner and whether this effect differed across task contexts and groups. The full model significantly improved model fit relative to the null model (χ²(7) = 143.91, *p* < 0.001). Because the model used a log link, coefficients represent changes in the log of expected press counts.

**Fig. 6:**
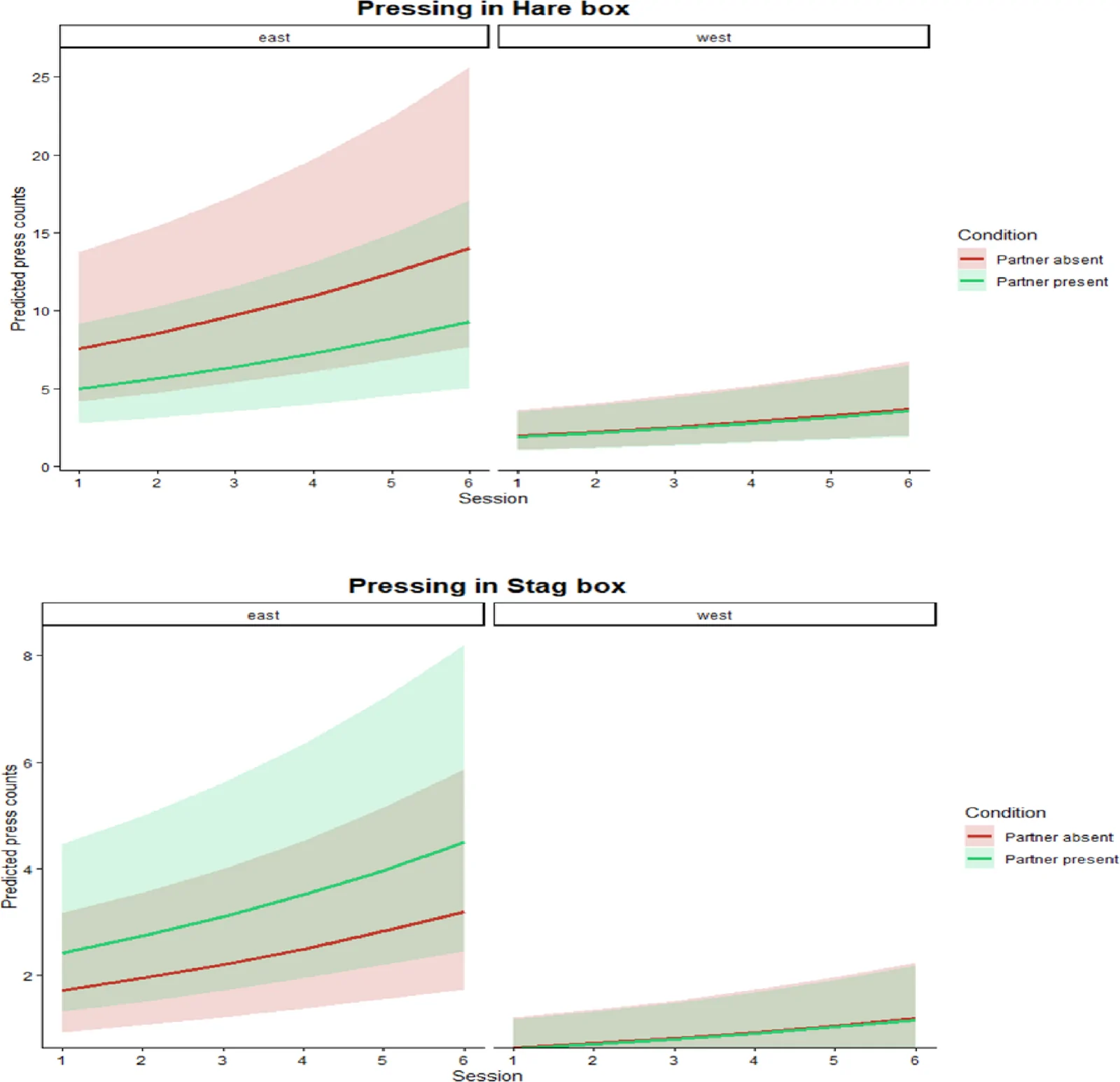
Predicted press counts across sessions in the Hare boxes (top row) and Stag boxes (bottom row) for the East and West groups. Lines represent model-predicted values from the generalized linear mixed model (GLMM), and shaded ribbons indicate 95% confidence intervals. Predictions are shown for conditions in which a partner was absent (red) and present (green). The model included partner presence, box type (Hare vs. Stag), group identity (East vs. West), and session, together with their interaction terms, with subject identity included as a random intercept to account for repeated observations across sessions

Importantly, the interaction between partner presence, box type and group was significant (β = −0.743 ± 0.355 SE, z = − 2.10, *p* = 0.036), indicating that the effect of partner presence on pressing behaviour depended on both task context and group identity. Estimated marginal means were therefore examined to interpret this interaction (Table 2; Fig. 6).

**Table 2:** Estimated marginal means of predicted press counts across partner conditions within box type and group

Box type	Group	Partner absent (mean ± SE)	Partner present (mean ± SE)	Δ Partner effect	95% CI absent	95% CI present
Hare	East	10.32 ± 3.08	6.84 ± 2.06	−3.48	[5.75, 18.52]	[3.80, 12.33]
Hare	West	2.71 ± 0.82	2.62 ± 0.79	−0.09	[1.50, 4.89]	[1.45, 4.73]
Stag	East	2.35 ± 0.71	3.30 ± 1.00	+ 0.96	[1.29, 4.26]	[1.83, 5.96]
Stag	West	0.88 ± 0.28	0.86 ± 0.27	−0.02	[0.47, 1.62]	[0.46, 1.58]

ᵃValues represent estimated marginal means (± SE) on the response scale

ᵇEstimates were derived from the best-fitting negative binomial generalized linear mixed model with log link

ᶜThe model included partner presence, box type (Hare vs. Stag), group identity (East vs. West), and session, with subject identity included as a random intercept to account for repeated observations across sessions

ᵈΔ Partner effect represents the difference between predicted means for the partner-present and partner-absent conditions within each box type and group

Pressing increased significantly across sessions (β = 0.124 ± 0.028 SE, z = 4.48, *p* < 0.001), indicating increasing utilisation of the apparatus over time (Table S4). The constituent effects of partner presence, box type and group were not interpreted independently as the interaction between partner presence, box type and group was significant. Instead, estimated marginal means and pairwise contrasts were used to examine the effect of partner presence within each combination of task context and group.

In the East group, partner presence influenced pressing behaviour differently across task contexts. In the cooperative Stag box, predicted press counts were slightly higher when a partner was present (EMM = 3.30) than when individuals were alone (EMM = 2.35), corresponding to a reduction in expected press rates when partners were absent (rate ratio = 0.71, z = − 1.998, *p* = 0.046). In contrast, in the Hare boxes individuals pressed more frequently when alone (EMM = 10.32) than when a partner was present (EMM = 6.84), resulting in significantly higher expected press rates in the partner-absent condition (rate ratio = 1.51, z = 2.34, *p* = 0.019).

In the West group, pressing behaviour remained comparatively low overall and showed little difference between partner-present and partner-absent conditions in either task context. In the Stag box, predicted press counts were similar when individuals were alone (EMM = 0.88) and when a partner was present (EMM = 0.86; rate ratio = 1.03, z = 0.12, *p* = 0.901). Similarly, in the Hare boxes press counts did not differ significantly between partner-present (EMM = 2.62) and partner-absent conditions (EMM = 2.71; rate ratio = 1.04, z = 0.21, *p* = 0.836). Pairwise contrasts are reported in Table S5.

These patterns indicate that partner presence influenced engagement with the apparatus differently across contexts and groups, with the clearest context-dependent contrast occurring in the East group (Fig. 6).

### Session-level patterns within task contexts

As the global model provided the primary test of partner-presence effects across task contexts and groups, the following analyses were treated as secondary follow-up models examining whether partner-associated pressing changed across sessions within each group-by-task context. These models were interpreted descriptively as context-specific temporal trajectories rather than as independent tests of group or task differences.

### Stag box

In the East group, press counts in the Stag box remained relatively stable across sessions, with little difference between partner-present and partner-absent conditions (Fig. S2a). Neither partner presence (β = 0.211 ± 0.298 SE, z = 0.71, *p* = 0.480), session (β = −0.046 ± 0.054 SE, z = − 0.84, *p* = 0.399), nor their interaction (β = 0.056 ± 0.074 SE, z = 0.76, *p* = 0.450) significantly predicted pressing behaviour (Table S6).

In contrast, the West group showed evidence of a temporal change in the cooperative (Stag) context (Fig. S2b). The interaction between partner presence and session was significant (β = 0.317 ± 0.154 SE, z = 2.07, *p* = 0.039), indicating that press counts in the partner-present condition increased more rapidly across sessions than those in the partner-absent condition (Table S6).

### Hare boxes

In the East group, pressing in the Hare boxes tended to occur more frequently when individuals were alone than when a partner was present (Fig. S3a). However, neither partner presence (β = 0.074 ± 0.255 SE, z = 0.29, *p* = 0.772), session (β = 0.030 ± 0.044 SE, z = 0.67, *p* = 0.501), nor their interaction (β = −0.102 ± 0.066 SE, z = − 1.55, *p* = 0.121) significantly predicted pressing behaviour (Table S7).

Similarly, in the West group pressing in the Hare boxes increased slightly across sessions but did not differ significantly between partner-present and partner-absent conditions (partner presence: β = 0.045 ± 0.495 SE, z = 0.09, *p* = 0.927). Session effects showed a marginal but non-significant increase over time (β = 0.157 ± 0.085 SE, z = 1.86, *p* = 0.063), and the interaction between partner presence and session was not significant (β = −0.024 ± 0.124 SE, z = − 0.19, *p* = 0.846) (Table S7).

Overall, these session-level analyses revealed limited temporal change in partner-associated pressing behaviour, with the clearest trajectory occurring in the West group Stag box condition.

More broadly, engagement with the apparatus varied across task contexts, groups and sessions. The pattern in the West group was changing over time and might have stabilised if we had provided more sessions, but within the given sessions there was no evidence for sensitivity to partner presence. The pattern in the East group revealed a clear sensitivity to partner presence, with higher press rates in the cooperative contexts when the partner was present.

However, we observed no clear instances of the screened communicative behaviours, including gaze alternation, gestures, body orientation, approach signals, or recruitment-like behaviours, that could have functioned to initiate or sustain cooperation.

## Discussion

Capuchins successfully cooperated in a Stag Hunt–based task even when individually obtainable rewards (albeit of a lower quality) were available. Our task allowed individuals to choose freely between cooperative and solo options, enabling us to examine how dyadic social relationships and individual traits shaped cooperative engagement. While prior Stag Hunt studies have often operationalised cooperation through computer- or token-mediated games (Brosnan 2011; Smith and Brosnan 2022) or apparatus-based tests with preselected dyads and assigned partners (Bullinger et al. 2011.; Duguid et al. 2014), the present approach increased ecological relevance by allowing free partner choice for a cooperative option while simultaneously offering an individual option. This setup aimed to recreate naturalistic decision-making contexts in which cooperative and individual strategies compete with each other (Mendres and De Waal 2000).

Cooperative outcomes were structured by both social and individual factors. Dyadic tolerance predicted which pairs successfully cooperated, while exploratory tendency and age class influenced individual participation. Partner presence also affected pressing behaviour differently across task contexts and groups. In the East Group, partner presence was associated with increased pressing in the cooperative Stag context but reduced pressing in the solo Hare context, whereas in the West Group, engagement with the Stag box increased more gradually across sessions. These findings suggest that cooperation emerged from how individuals navigated the social demands of the task, including partner access, tolerance and willingness to engage with the apparatus. At the same time, it remains unclear whether similar patterns would emerge in wild settings, where individuals must coordinate under less predictable ecological and social conditions, including fluctuating food availability, greater spatial spread between group members, shifting partner availability, increased competition around resources, and the absence of stable or repeatedly accessible cooperative apparatuses (Mendres and De Waal 2000; Hirata and Fuwa 2007; Seed et al. 2008; Drea and Carter 2009; Brosnan 2011; Plotnik et al. 2011; Suchak et al. 2014; Molesti and Majolo 2016).

Social factors, specifically tolerance, helped explain which dyads successfully cooperated. Capuchins did not cooperate indiscriminately; dyads characterised by higher inter-individual tolerance were significantly more likely to engage in cooperative behaviour. This reinforces previous findings that tolerance is a critical prerequisite for successful cooperation (Melis et al. 2006a; Hare et al. 2007; Massen et al. 2015; Molesti and Majolo 2016). When tolerance and affinity were considered together, tolerance appeared to be the more important enabling factor. This pattern aligns with cross-species findings suggesting that the ability to tolerate proximity and share resources without conflict, rather than relationships quality, can determine whether cooperation occurs. For instance, chimpanzee pairs with higher levels of food-sharing tolerance were more successful in rope-pulling cooperation tasks (Melis et al. 2006a), and ravens preferentially cooperated with socially tolerant partners rather than long-term affiliates (Massen et al. 2015). Together, these findings highlight the importance of distinguishing between social affinity and functional tolerance in facilitating cooperation.

Alongside social tolerance, individual exploratory tendency influenced cooperative outcomes by shaping engagement with the task. In the open-group setting, all individuals were able to approach the apparatus, but participation depended on when and how they entered the task space. Apparatus occupancy, local crowding, displacement risk and rank-related social dynamics may, therefore, have affected opportunities to interact with the boxes. Consistent with research linking exploratory tendencies and reduced neophobia to engagement with novel contexts (Wilson and Sober 1994; Gilby et al. 2008; Scheid and Noë 2010; Molesti and Majolo 2016), more exploratory individuals may have engaged more readily during the tolerance and Hare phases, thereby gaining greater familiarity with the apparatus and, consequently, more opportunities to participate once the cooperative Stag option became available. Here, approach behaviour should be interpreted as reflecting both individual responsiveness to the apparatus and the social conditions governing access to it, rather than as a purely individual temperament effect. Similar patterns have been documented across species, where reduced neophobia facilitates engagement with novel problems (Kendal et al. 2005; Amici et al. 2019). Although exploratory individuals can sometimes influence the participation of others and indirectly facilitate cooperative interactions (Scheid and Noë 2010; Massen and Koski 2014; Molesti and Majolo 2016), such dyadic facilitation effects were not directly tested here.

Age composition, but not sex, also influenced cooperative outcomes in the East Group, where dyads involving juveniles contributed disproportionately to successful cooperative interactions. Age was not analysed as a predictor in the West Group because the group consisted exclusively of adults. This age-dependent pattern may reflect reduced competitive tension around juveniles, greater behavioural flexibility, or increased adult tolerance in adult–juvenile combinations (Molesti and Majolo 2016). Moreover, juvenile primates often display higher levels of exploration and lower neophobia than adults, which can facilitate engagement with novel tasks (Kendal et al. 2005). Such factors may increase the likelihood of successful coordination. When considered alongside the adult-only composition of the West Group, these findings suggest that developmental diversity may enhance cooperative flexibility within social groups.

Although simultaneous lever pressing in the Stag Box does not necessarily require complex cognitive representations of a partner’s role (Schmelz et al. 2017), our data provide some insight into whether capuchin individuals modulate engagement in relation to partner availability. Cooperative performance in this paradigm should, therefore, be interpreted as evidence of successful coordination under permissive social conditions rather than definitive proof of role-based task understanding. In the East Group, the contrast between increased pressing in the Stag context when a partner was present and increased pressing in the Hare context when alone may suggest some level of sensitivity to the differing functional demands of cooperative and individual options. However, alternative explanations remain plausible.

Nevertheless, these patterns cannot be dismissed as purely incidental. Younger individuals often exhibit greater behavioural flexibility and exploratory learning than adults, which can facilitate the discovery of novel action–outcome contingencies (Gopnik et al. 2004; Kendal et al. 2005; Amici et al. 2019). Repeated interactions by juveniles may, therefore, have facilitated learning both individually and potentially through social facilitation or observation by other group members. The higher number of cooperative bouts in the East Group may also have provided more opportunities for individuals for learning of the contingency between partner presence and success. Under this interpretation, the contrast between Stag and Hare contexts could reflect emerging discrimination between cooperative and solo options, although the present group-testing design does not allow these alternatives to be clearly distinguished.

Session-level analyses provided further insight into these group differences in partner-associated pressing behaviour. In the East Group, partner-associated pressing patterns appeared relatively stable across sessions, whereas in the West Group, partner-associated pressing in the Stag context increased over time. This pattern may reflect increasing familiarity with the apparatus or repeated cooperative successes arising through social facilitation. Notably, this trajectory occurred only in the Stag Box and not in the Hare Boxes, suggesting that changes in engagement were specific to the cooperative context. With additional testing sessions, a clearer behavioural divergence between cooperative (Stag) and non-cooperative (Hare) contexts may potentially have emerged.

Kinship and dominance are well-established factors influencing cooperation in primates (Suchak et al. 2014). However, neither variable was included in the present analysis due to limited variability in the dataset. Most individuals within each group were closely related, restricting the ability to statistically assess kinship effects. Similarly, dominance hierarchies were not sufficiently differentiated, particularly in the West Group, to allow reliable classification into rank categories. Within the recorded trials there were also no clear instances of communicative behaviours such as gaze alternation, gestures, or recruitment behaviours that have been documented in other cooperative coordination tasks (Leavens et al. 2005; Melis et al. 2006b).

More broadly, these findings contribute to a growing body of work suggesting that cooperative outcomes in group-living animals often emerge from the interaction between social compatibility, individual behavioural tendencies, and the nature of cooperative tasks rather than from specific mechanisms for social coordination. When tolerant partners repeatedly encounter cooperative opportunities within flexible social environments, coordinated behaviour may arise through repeated interaction, behavioural alignment, and learning processes. In this sense, cooperation in capuchins may reflect the convergence of social tolerance, developmental diversity, and task affordances that together create conditions under which coordination can emerge.

Future work could more directly examine partner sensitivity using designs that require individuals to actively wait for a partner, a capacity demonstrated in several species, including ravens, elephants, canids and chimpanzees (Plotnik et al. 2011; Asakawa-Haas et al. 2016; Marshall-Pescini et al. 2017; Range et al. 2019). It would also be possible to allow individuals to choose explicitly between cooperative and individual apparatuses. In the present study, context-dependent behavioural modulation in relation to partner presence was observed in one group, with changes in pressing behaviour occurring specifically in the cooperative Stag context and not in the solo Hare context. This context specificity is important because indiscriminate increases in pressing across both apparatuses could instead reflect general social facilitation rather than adjustment to the cooperative demands of the task. Within the framework proposed by Duguid and Melis (2020), the present findings appear to fall between socially influenced cooperation and the early emergence of more actively coordinated forms of collaboration. The context-dependent behavioural adjustment observed further raises the possibility that capuchins may be capable of more actively coordinated cooperation under certain conditions. However, distinguishing these possibilities will require more explicit experimental testing of whether individuals intentionally modify their behaviour according to a partner’s role or availability. Future studies should therefore investigate behaviours such as waiting for partners, recruitment attempts, communicative signalling, and flexible adjustment to partner-dependent contingencies, while also exploring whether capuchins show any evidence of shared intentionality during cooperative interactions. In this respect, the present findings contrast with earlier capuchin studies reporting little or no sensitivity to partner roles (Chalmeau et al. 1997; Visalberghi et al. 2000), while remaining broadly consistent with Mendres and de Waal’s observation that capuchins can show partner-related behavioural adjustment in cooperative situations (Mendres and De Waal 2000).

In conclusion, capuchin monkeys successfully engaged in cooperation in a Stag Hunt framework that simultaneously provided multiple individual non-cooperative alternatives and unrestricted partner choice. Cooperative outcomes were shaped both by dyadic social relationships and individual behavioural tendencies, with tolerance strongly predicting which dyads cooperated and individual exploratory tendency influencing engagement with the task. The disproportionate involvement of juveniles further suggests that developmental diversity within social groups may facilitate the emergence of cooperative interactions by increasing exploratory engagement and reducing competitive constraints. Although the present design cannot conclusively demonstrate causal understanding of a partner’s role, behavioural patterns across contexts indicate that partner availability can influence engagement with cooperative opportunities under certain conditions. The present findings highlight how cooperation in socially tolerant primates may arise from the interaction between social compatibility, individual behavioural variation, and the affordances of the task environment. Experimental paradigms that allow free partner choice and meaningful alternatives therefore provide a valuable framework for understanding how cooperative behaviour emerges within socially dynamic groups.

## Electronic Supplementary Material

Below is the link to the electronic supplementary material.

Supplementary file 1

## Data Availability

The datasets and code scripts supporting the analyses reported in this study are available in a private Open Science Framework repository via the following view-only link: https://osf.io/dnx9b/overview? view_only=fb2e44a74e0444d290eae0d629221431 . Raw video recordings are available from the corresponding author upon reasonable request and subject to institutional and ethical approval.

## Materials availability

Materials or apparatus used in this study are available from the corresponding author on reasonable request.
