# Supplementary material for: Cooperation in brown capuchin monkeys: social tolerance, individual traits, and partner sensitivity in a Stag Hunt task
Source: Anim Cogn. 2026 Sep 16;29(1):73. doi: 10.1007/s10071-026-02107-8 (PMC13695629; doi:10.1007/s10071-026-02107-8)
Supplement: Supplementary file 1 [file 10071_2026_2107_MOESM1_ESM.docx]

**Supplementary Information:**

**Fig. S1** Cumulative participation and interaction patterns at the Stag box across sessions in the East and West groups.
(a) Cumulative number of unique dyads recorded with overlapping presence at the Stag box across sessions. A dyad was counted as present together only when the two individuals’ presence intervals at the Stag box overlapped within the same session, (b) Cumulative number of unique dyads that successfully cooperated at the Stag box at least once across sessions,
(c) Number of individuals first recorded as successful cooperators at the Stag box in each session.

**Table S1.** Results of the combined binomial generalized linear mixed model examining the effects of dyadic tolerance, affiliative bond strength, group identity and their interactions on the likelihood of dyadic cooperation in brown capuchin monkeys.

| **Predictor** | **Estimate** | **SE** | **z** | **p** |
| --- | --- | --- | --- | --- |
| Intercept | 3.438 | 8.838 | 0.389 | 0.697 |
| **Tolerance** | **1.701** | **0.535** | **3.182** | **0.001** |
| Affinity | 12.503 | 18.836 | 0.664 | 0.507 |
| Group West | -7.981 | 8.932 | -0.893 | 0.372 |
| Tolerance x Group West | -0.356 | 0.856 | -0.416 | 0.677 |
| Affinity x Group West | -11.720 | 18.833 | -0.622 | 0.534 |

*ᵃ Binomial generalized linear mixed model with a logit link and crossed random intercepts for dyad members (ID1 and ID2). Significant outcomes are shown in bold.*

*ᵇ Tolerance and affinity were mean-centred and z-standardised across the combined dataset prior to analysis.*

*ᶜ The East group was treated as the reference category for group identity.*

*ᵈ Predictor-specific significance was evaluated using likelihood-ratio tests comparing the full model with reduced models in which each predictor (or interaction term) was removed while retaining the remaining predictors. The table reports model coefficients from the full model.*

**Table S2** Results of negative binomial GLMs examining individual predictors of cooperative participation.

| **Predictor** | **Estimate (East)** | **SE** | **z** | **p** | **Estimate (West)** | **SE** | **z** | **p** |
| --- | --- | --- | --- | --- | --- | --- | --- | --- |
| Intercept (Adult Female ref) | **−2.312** | **1.031** | **−2.24** | **0.025** | −1.004 | 0.871 | −1.15 | 0.249 |
| Exploratory tendency | **0.343** | **0.076** | **4.52** | **<0.001** | **0.313** | **0.093** | **3.38** | **0.001** |
| Age (Infant) | −1.529 | 1.277 | −1.20 | 0.232 | — | — | — | — |
| Age (Juvenile) | **1.880** | **0.769** | **2.44** | **0.014** | — | — | — | — |
| Sex (Male) | 0.237 | 0.702 | 0.33 | 0.736 | −1.143 | 0.769 | −1.49 | 0.137 |

*ᵃ Negative binomial GLM with log link.* *Significant outcomes are shown in bold.
ᵇ Reference category for age was adult.
ᶜ Reference category for sex was female.
ᵈ Predictor-specific significance was evaluated using likelihood-ratio tests comparing the full model with reduced models in which each predictor was removed while retaining the remaining predictors. The table reports model coefficients from the full negative binomial models.*

**Table S3** Results of negative binomial GLMMs examining the effects of exploratory tendency and session on overall task engagement counts in the East and West groups.

| **Group** | **Predictor** | **β** | **SE** | **z** | **p** | **IRR** | **95% CI for IRR** |
| --- | --- | --- | --- | --- | --- | --- | --- |
| **East** | **Intercept** | **2.055** | **0.304** | **6.765** | **<0.001** | 7.808 | 4.305–14.163 |
| **East** | **Exploratory tendency** | **1.327** | **0.318** | **4.169** | **<0.001** | 3.770 | 2.020–7.036 |
| East | Session | 0.031 | 0.051 | 0.606 | 0.544 | 1.031 | 0.934–1.139 |
| **West** | **Intercept** | **2.195** | **0.234** | **9.370** | **<0.001** | 8.984 | 5.676–14.220 |
| **West** | **Exploratory tendency** | **0.571** | **0.226** | **2.531** | **0.011** | 1.771 | 1.138–2.757 |
| **West** | **Session** | **0.431** | **0.069** | **6.221** | **<0.001** | 1.539 | 1.344–1.763 |

*ᵃ Negative binomial GLMM with a log link. Overall task engagement counts were modelled as a function of exploratory tendency and session separately for the East and West groups.*

*ᵇ Subject identity was included as a random intercept to account for repeated observations across sessions.*

*ᶜ Exploratory tendency and session were standardised within each group before analysis.*

*ᵈ Positive coefficients indicate increases in expected engagement counts on the log scale; exponentiated coefficients are reported as incidence-rate ratios.*

*ᵉ Significant outcomes (p < 0.05) are shown in bold.*

*ᶠ Overall task engagement was defined as the total number of press and touch behaviours directed towards all experimental boxes within a session.*

**Table S4** Results of the full generalized linear mixed model (GLMM) examining whether press counts differed according to partner presence, box type (Hare vs. Stag), group identity (East vs. West), and session.

| **Predictor** | **β** | **SE** | **z** | **p** |
| --- | --- | --- | --- | --- |
| **Intercept** | **2.334** | **0.298** | **7.822** | **<0.001** |
| **Partner presence (present)** | **−0.411** | **0.176** | **−2.338** | — |
| **Box type (Stag)** | **−1.481** | **0.186** | **−7.955** | — |
| **Group (West)** | **−1.338** | **0.424** | **−3.156** | — |
| **Session** | **0.124** | **0.028** | **4.476** | **<0.001** |
| **Partner × Box** | **0.753** | **0.245** | **3.076** | — |
| Partner × Group | 0.377 | 0.240 | 1.570 | — |
| Box × Group | 0.354 | 0.263 | 1.346 | — |
| **Partner × Box × Group** | **−0.743** | **0.355** | **−2.095** | **0.036** |

*ᵃ Negative binomial GLMM with log link. Press counts were modelled as a function of partner presence, box type, group identity, and session, including all interaction terms.*

*ᵇ Subject identity was included as a random intercept to account for repeated observations across sessions.*

*ᶜ The reference categories were partner absent, Hare box, and East group.*

*ᵈ Positive coefficients indicate increases in expected press counts on the log scale; negative coefficients indicate decreases relative to the reference category.*

*ᵉ Where reported, significant outcomes (p < 0.05), are shown in bold.*

*ᶠ P-values are not shown for constituent main effects and lower-order interactions involved in the significant Partner presence × Box type × Group interaction because these coefficients represent conditional effects evaluated at the reference levels of the other interacting predictors and are not interpretable as general effects.*

**Table S5** Pairwise contrasts from estimated marginal means comparing press counts between partner-absent and partner-present conditions within each box type and group.

| **Box type** | **Group** | **RR (Absent/Present)** | **SE** | **z** | **p** |
| --- | --- | --- | --- | --- | --- |
| **Hare** | **East** | **1.51** | **0.265** | **2.34** | **0.019** |
| **Stag** | **East** | **0.71** | **0.121** | **−2.00** | **0.046** |
| Hare | West | 1.04 | 0.170 | 0.21 | 0.836 |
| Stag | West | 1.03 | 0.205 | 0.12 | 0.901 |

*ᵃ Pairwise contrasts were derived from estimated marginal means of the negative binomial GLMM with log link.*

*ᵇ Rate ratios (RR) represent the ratio of predicted press counts in the partner-absent condition relative to the partner-present condition within each box type and group.*

*ᶜ RR > 1 indicates higher predicted press counts when individuals were alone, whereas RR < 1 indicates higher predicted press counts when a partner was present.*

*ᵈ Significant results (p < 0.05) are shown in bold.*

Fig. S2a	Fig. S2b

**Fig. S2** Press counts across sessions under partner-present and partner-absent conditions in the Stag box. Predicted press counts (± 95% confidence intervals) are shown across sessions for capuchins in the East (Fig. S1a) and West (Fig. S1b) groups. Lines represent model-estimated values from generalized linear mixed models with negative binomial (East: zero-inflated negative binomial; West: negative binomial) error structures. Shaded areas indicate model-based confidence intervals. Predictions are plotted separately for partner-present and partner-absent conditions to assess sensitivity to a partner’s role across repeated sessions.

**Table S6** Results of negative binomial GLMMs examining effects of partner presence and session on Press counts in the Stag box.

| **Predictor** | **Estimate (East)** | **SE** | **z** | **p** | **Estimate (West)** | **SE** | **z** | **p** |
| --- | --- | --- | --- | --- | --- | --- | --- | --- |
| Intercept | **1.047** | **0.457** | **2.29** | **0.022** | −0.411 | 0.483 | −0.85 | 0.395 |
| Partner presence | 0.211 | 0.298 | 0.71 | 0.480 | **−1.347** | **0.631** | **−2.13** | **—** |
| Session | −0.046 | 0.054 | −0.84 | 0.399 | 0.132 | 0.103 | 1.28 | — |
| Partner presence × Session | 0.056 | 0.074 | 0.76 | 0.450 | **0.317** | **0.154** | **2.07** | **0.039** |

*ᵃ Negative binomial GLMM with log link.* *Significant outcomes are shown in bold.
ᵇ Subject identity included as a random intercept.
ᶜ Session treated as a continuous predictor.
ᵈ The East Group model included a zero-inflation component.*

*ᵉ For the West-group model, p-values are not shown for partner presence and session because their interaction was significant. These coefficients therefore represent effects conditional on the value of the interacting predictor rather than general effects.*

Fig. S3a	Fig. S3b

**Fig. S3** Press counts across sessions under partner-present and partner-absent conditions in the Hare box. Predicted press counts (± 95% confidence intervals) are shown across sessions for capuchins in the East (Fig S2a) and West (Fig S2b) groups. Lines represent model-estimated values from generalized linear mixed models with negative binomial error structures. Shaded areas indicate model-based confidence intervals. Predictions are plotted separately for partner-present and partner-absent conditions to evaluate whether pressing behaviour in the solo (Hare) context varied as a function of partner presence over repeated sessions.

**Table S7** Results of negative binomial GLMMs examining effects of partner presence and session on Press counts in the Hare box.

| **Predictor** | **Estimate (East)** | **SE** | **z** | **p** | **Estimate (West)** | **SE** | **z** | **p** |
| --- | --- | --- | --- | --- | --- | --- | --- | --- |
| Intercept | **2.100** | **0.263** | **8.177** | **<0.001** | 0.262 | 0.510 | 0.514 | 0.607 |
| Partner presence | -0.05 | 0.255 | -0.199 | 0.842 | 0.045 | 0.495 | 0.091 | 0.927 |
| Session | 0.017 | 0.044 | 0.386 | 0.7 | 0.157 | 0.085 | 1.861 | 0.063 |
| Partner presence × Session | -0.074 | 0.066 | −1.143 | 0.253 | −0.024 | 0.124 | −0.194 | 0.846 |

*ᵃ Negative binomial GLMM with log link.* *Significant outcomes are shown in bold.
ᵇ Subject identity included as a random intercept.
ᶜ Session treated as a continuous fixed predictor.*
